# Testing magnetic resonance imaging parameters to predict pituitary macroadenoma consistency

**DOI:** 10.1055/s-0045-1813239

**Published:** 2025-12-02

**Authors:** Thaylla Maybe Bedinot da Conceição, Jaisa Quedi Araújo da Silva, Matheus de Lima Ruffini, Adolfo Moraes de Souza, Fabiano Reis, Ápio Cláudio Martins Antunes, Marino Muxfeldt Bianchin, Juliana Ávila Duarte

**Affiliations:** 1 Hospital de Clínicas de Porto Alegre, Porto Alegre RS, Brazil.; 2 Universidade Federal do Rio Grande do Sul, Faculdade de Medicina, Departamento de Medicina Interna, Porto Alegre RS, Brazil.; 3 Universidade Estadual de Campinas, Faculdade de Ciências Médicas, Departamento de Clínica Médica, Campinas SP, Brazil.

**Keywords:** Pituitary Neoplasms, Magnetic Resonance Imaging, Neuroimaging, Surgical Oncology

## Abstract

**Background:**

Pituitary macroadenomas (PMAs) are frequently encountered tumors, predominantly characterized as soft and easily resectable during neurosurgery. In contrast, fibrous PMAs present difficulties during surgical removal. Therefore, the ability to predict the consistency of PMAs preoperatively could enhance surgical planning.

**Objective:**

To evaluate the ability of conventional T2-weighted imaging (T2WI) to predict PMA consistency by comparing isolated tumor signal intensity with the adenoma-to-middle cerebellar peduncle (ACP) ratio.

**Methods:**

Magnetic resonance imaging (MRI) scans from 45 patients with PMAs were independently reviewed by 3 blinded radiologists. For each case, the signal intensity (SI) of the adenoma and of the middle cerebellar peduncle was measured, and the ACP ratio (SI _adenoma_ /SI _peduncle_ ) was calculated. Tumor consistency (soft or fibrous) was determined intraoperatively by a single neurosurgeon.

**Results:**

Intraoperative assessment classified 29 PMAs (64.4%) as soft and 16 (35.6%) as fibrous. Isolated adenoma SI on T2WI differed significantly between soft and fibrous tumors ( *p* = 0.013), while the ACP ratio demonstrated stronger discriminatory power ( *p* < 0.0001). The receiver operating characteristic (ROC) curve yielded an area under the curve of 0.939 for the ACP ratio. Threshold values > 1.59 were highly predictive of soft tumors (sensitivity 72.4%; specificity 100.0%), whereas values < 1.27 were associated with fibrous tumors (sensitivity 100.0%; specificity 37.5%).

**Conclusion:**

Although isolated adenoma SI on T2WI showed statistical significance, it was not sufficient for consistent preoperative prediction of tumor consistency. The ACP ratio provided superior accuracy and clinical utility, supporting its role as a noninvasive imaging biomarker to enhance preoperative assessment and surgical planning in patients with pituitary macroadenomas.

## INTRODUCTION

Most pituitary macroadenomas (PMAs) are soft, facilitating effective transsphenoidal resection. However, ∼ 10% of these tumors are fibrous, which may affect the surgical approach. Various magnetic resonance imaging (MRI) techniques have been explored to ascertain PMA consistency, including the evaluation of standard T1 and T2 signals, contrast enhancement characteristics, diffusion, and elastography. 1 2 3 4 5 6 7 8 9 Some studies 1 3 10 11 12 13 14 15 16 17 18 19 have attempted to correlate MRI findings with tumor consistency based on water content, but results have varied. While certain studies 1 3 10 11 12 successfully predicted PMA consistency, others 13 14 15 16 17 did not.

In the present study, we sought to validate the parameters proposed by Smith et al. 1 for the preoperative assessment of pituitary macroadenoma consistency. The middle cerebellar peduncle was selected as an internal reference standard for T2-weighted signal intensity (SI), owing to its anatomical stability and reproducibility. Based on this reference, we calculated the adenoma-to-peduncle (ACP) ratio and investigated its value as a quantitative predictor of tumor consistency, with the aim of enhancing preoperative diagnostic accuracy and supporting surgical decision-making in patients with pituitary macroadenomas.

## METHODS

In the present investigation, we attempted to reproduce the study conducted by Smith et al. 1 to assess the reproducibility of their findings under similar conditions. By applying the same parameters and methodologies described in their work, we aimed to verify whether the outcomes observed in their study could be replicated in our dataset.

Following approval from the Hospital's Ethics Committee (N° CAAE: 62407716.0.0000.5327), our study included 45 patients diagnosed with macroadenomas (size > 1.0 cm), admitted to Hospital de Clínicas de Porto Alegre, Porto Alegre, state of Rio Grande do Sul, Brazil, between January 2010 and January 2018. All patients underwent endoscopic endonasal transsphenoidal surgery performed by a single surgeon. During surgery, PMA consistencies were determined and classified as either soft (if easily removed through curettage and aspiration) or fibrous (if suction was ineffective and required techniques such as bipolar electrocoagulation, ultrasonic aspiration, or sharp segmentation for removal). Although this classification is subjective, it is widely adopted by neurosurgeons. The present study received approval from the Research Ethics Committee (protocol n.160586) and adhered to the principles of the Declaration of Helsinki.

### Neuroimaging

Magnetic resonance imaging was conducted using either a 1.5 Tesla Achieva MR scanner (41 patients) or a 3.0 Tesla Ingenia MR scanner (4 patients) (both from Philips Medical Systems). Initially, unenhanced images were obtained with axial T2-weighted imaging (T2WI) (RT/ET [repetition time/echo time] = 5,257/100 ms; matrix = 0.599 × 0.75; FOV [field of view] = 220 × 170 × 155 mm; section thickness/gap = 5/1.0 mm), coronal T1-weighted imaging (T1WI) (RT/ET = 400–700/15 ms; section thickness/gap = 2.5/0.3 mm; FOV = 120 × 120 × 33; matrix = 0.74 × 0.92), and coronal T2WI (RT/ET = 2,000–4,000/100 ms; matrix = 0.57 × 0.71; FOV = 120 × 120 × 33 mm; section thickness/gap = 2.5/0.3 mm). Subsequently, coronal T1-dynamic fast spin-echo (FSE) imaging (RT/ET = 400–700/15 ms; FOV= 120 × 120 × 33; matrix = 0.67 × 0.84) was performed following a bolus injection of contrast media (gadopentetate dimeglumine, 0.1 ml/kg). The conventional coronal T1WI FSE sequence was repeated immediately after the dynamic scan. All scans had a slice thickness of 2.5 mm and a gap of 0.3 mm. Additionally, MRI examinations of 4 patients were completed on the 3.0 Tesla scanner with standard T1WI and T2WI images, as well as gadolinium-enhanced T1WI targeting the pituitary gland. Initially, imaging without enhancement was performed using axial T2WI (RT/ET = 3,000/80 ms; matrix = 576 × 576 × 576; FOV = 230 × 185 × 139 mm; section thickness/gap = 4.00/1.00 mm), coronal T1WI (RT/ET = 300/15, section thickness/gap = 2.5/0.3 mm; FOV = 110/110; matrix = 156 × 140), and coronal T2WI (RT/ET = 3,000/90 ms; matrix = 200 × 172; FOV = 110 × 110 × 33 mm; section thickness/gap = 2.0/0.2 mm). Following this, dynamic FSE imaging (RT/ET = 986/12 ms; section thickness/gap = 2.5/0.25 mm; FOV = 110 × 110 mm; matrix = 112 × 110) began after bolus injection of contrast material (gadopentetate dimeglumine, 0.1 ml/kg) (GE Healthcare. Six sets of dynamic images, each comprising 3 slice locations, were acquired at 24-second intervals. The conventional coronal T1WI FSE sequence was repeated immediately after the dynamic scan. All scans had a slice thickness of 2.5 mm and a gap of 0.25 mm.

Using the T2WI sequence, we calculated a ratio based on the PMAs' signal and middle cerebellar peduncle measurements, as proposed by Smith et al. 1 Three board-certified radiologists, blinded to intraoperative tumor consistency perceptions and histopathological diagnoses, independently evaluated all neuroimaging and T2WI sequences within the adenomas, cerebellar peduncles, and ACP ratios. In case of discrepancies, a consensus was reached.

**Figure 1:**
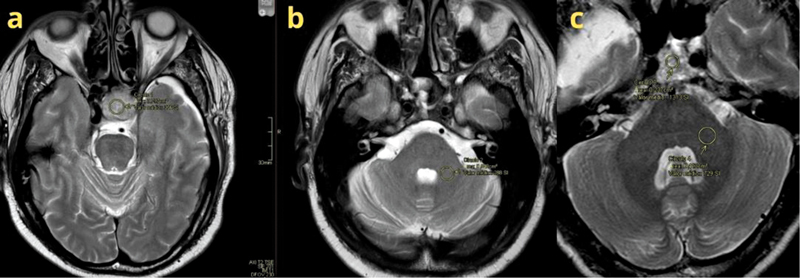
Exemplification of region of interest (ROI) placement for the adenoma-to-cerebellar peduncle ratio (ACP). Magnetic resonance imaging T2-weighted images of patients #1 and #2. 1a) Circle 1–ROI positioned within the homogeneous macroadenoma of patient #1. 1b) Circle 2–ROI positioned in the middle cerebellar peduncle of patient #1. 1c) Circles 3 and 4–ROIs exemplifying placement within the heterogeneous macroadenoma (circle 3) and the middle cerebellar peduncle (circle 4) of patient #2.

A region of interest (ROI) was meticulously delineated on T2WI using an electronic cursor ( Figure 1 ). Measurements were obtained from the solid components of the tumors exhibiting the lowest signal intensity (SI) on T2WI, while excluding necrotic and hemorrhagic areas. Three distinct ROIs were selected within the adenoma to ensure homogeneous sampling of intensity, and the mean intensity value was calculated from these ROIs for each tumor. Subsequently, a second ROI was positioned at the middle cerebellar peduncle, serving as a consistent anatomical reference, selected for its uniform distribution of MRI SI, thereby minimizing potential measurement errors. The ACP ratio was then calculated as SI _adenoma_ /SI _peduncle_ .

### Statistics

Categorical variables were compared using a 2-tailed Fisher's exact test, with results expressed as odds ratios (ORs) and 95% confidence intervals (CIs). Quantitative variables were compared using the Student t-test for independent samples, with results expressed as mean (± standard deviation [SD]). A receiver operating characteristic (ROC) curve was generated to ascertain sensitivity, specificity, and accuracy of the MRI T2WI sequence ratio between adenoma and cerebellar peduncle signal measurements in predicting PMA consistency. Furthermore, we compared our sample's ROC curve with one derived from the results of Smith et al. 1 As the results were not statistically different, we combined both datasets for a more representative analysis. A *p* -value < 0.05 was deemed statistically significant.

## RESULTS

The clinical variables of the patients are summarized in Table 1 , and Table 2 details individual patient results. No significant differences were noted between tumor classes regarding demographic or clinical variables, such as biological sex, age, contrast enhancement patterns, and completeness of surgical treatment. Initially, all patients included in the present study underwent endoscopic endonasal surgery. Out of the participants, 25 (55%) were female, with an average age of 56 years old (SD = 13.25). In terms of tumor classification, 29 (64.4%) were categorized as soft consistency, while 16 (35.5%) were identified as fibrous consistency. Neuroimaging was assessed by a radiologist using postcontrast T1WI to determine homogeneity or heterogeneity. It was found that 23 tumors (51.1% of the total sample) were homogeneous in relation to gadolinium contrast enhancement. Additionally, anatomical and immunohistochemical analyses identified cell types, with 28 tumors (62.2%) producing pituitary hormones.

**Table 1:** Clinical variables according to pituitary macroadenomas' consistency

		Total ( *n =* 45)	Soft ( *n* = 29)	Fibrous ( *n* = 16)	OR (95%CI)	*p-value*
**Age,** years old (SD)		56.63 (13.25)	56.10 (12.95)	57.31 (14.16)		0.773(#)
**Sex**	Male	20 (45.5%)	12 (41.4%)	8 (50%)	1.42 (0.41–4.83)	0.755(*)
	Female	25 (55.5%)	17 (58.6%)	8 (50%)		
**Enhancement**	Homogeneous	23 (51.1%)	15 (51.7%)	8 (50%)	1.07 (0.32–3.63)	1.000(*)
	Heterogeneous	22 (48.9%)	14 (48.3%)	8 (50%)		
**Immunohistochemistry**	Secretor	28 (62.2%)	18 (62%)	10 (62.5%)	0.98 (0.28–3.46)	1.000(*)
	Nonsecretor	17 (37.8%)	11 (38%)	6 (37.5%)		
**Surgery**	Complete	26 (57.8%)	19 (65.5%)	7 (43.7%)	2.44 (0.7–8.52)	0.212(*)
	Incomplete	19 (42.2%)	10 (34.5%)	9 (56.3%)		

Abbreviations: CI, confidence interval; OR, odds ratio; SD, standard deviation.

Notes: (#) Student t-test; (*) Fischer's exact test.

**Table 2:** Descriptions of the 45 patients

Patient	Sex	Age (years old)	Index of ACP	Neurosurgery consistency
1	F	69	1.63	Soft
2	F	39	1.50	Soft
3	F	74	1.61	Soft
4	M	67	1.74	Soft
5	F	37	1.64	Soft
6	M	59	1.45	Fibrous
7	M	56	1.89	Soft
8	M	81	1.51	Soft
9	F	65	1.68	Soft
10	F	50	1.30	Soft
11	F	42	1.50	Soft
12	M	47	1.62	Soft
13	M	72	1.84	Soft
14	M	54	0.98	Fibrous
15	M	66	1.13	Fibrous
16	F	56	1.61	Soft
17	M	73	1.25	Fibrous
18	F	54	1.98	Soft
19	M	58	1.41	Fibrous
20	F	67	1.49	Fibrous
21	F	52	1.66	Soft
22	F	48	1.64	Soft
23	M	75	1.46	Fibrous
24	F	49	2.03	Soft
25	F	59	1.40	Soft
26	M	40	1.60	Soft
27	F	64	1.56	Soft
28	F	53	1.71	Soft
29	M	43	1.56	Soft
30	F	24	1.36	Fibrous
31	M	77	1.76	Soft
32	M	72	1.57	Fibrous
33	M	80	1.70	Soft
34	F	64	1.68	Soft
35	F	43	1.83	Soft
36	F	39	1.58	Soft
37	M	43	1.66	Soft
38	F	58	1.32	Fibrous
39	F	63	1.32	Fibrous
40	F	55	1.40	Fibrous
41	F	64	1.00	Fibrous
42	M	35	1.00	Fibrous
43	M	57	1.53	Soft
44	F	54	0.90	Fibrous
45	M	46	1.60	Soft

Abbreviations: ACP, adenoma-to-cerebellar peduncle ratio; F, female; M, male.

**Figure 2:**
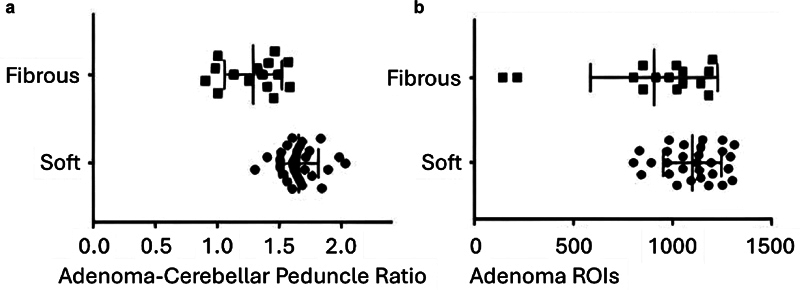
Distribution of signal intensity based on macroadenoma consistency. a) Distribution of the crude signal intensity of pituitary macroadenomas (PMAs) according to consistency. The mean signal intensity of ROIs for fibrous and soft PMAs revealed a statistically significant difference ( *p* = 0.013). b) Signal intensity ratio of adenoma-to-cerebellar peduncle (ACP) for PMAs based on consistency. The mean ACP signal intensity ratio of ROIs for fibrous and soft PMAs also demonstrated a statistically significant difference ( *p* < 0.0001).

Of the total study sample, 26 (57.8%) pituitary tumors were completely excised during transsphenoidal surgery, while 19 patients (10 with soft tumors and 9 with fibrous tumors) did not achieve complete tumor removal, primarily due to cavernous sinus invasion. In terms of neuroimaging studies, the isolated T2WI of the PMAs and the ACP ratio effectively distinguished tumor consistency in the preoperative phase, with the latter demonstrating superior accuracy. The average ACP ratio was 1.654 (SD = 0.157) for the 29 soft tumors and 1.287 (SD = 0.222) for the 16 fibrous tumors, with a *p* -value < 0.0001 (95%CI: 0.795–0.977). These results are illustrated in Figure 2 .

**Figure 3:**
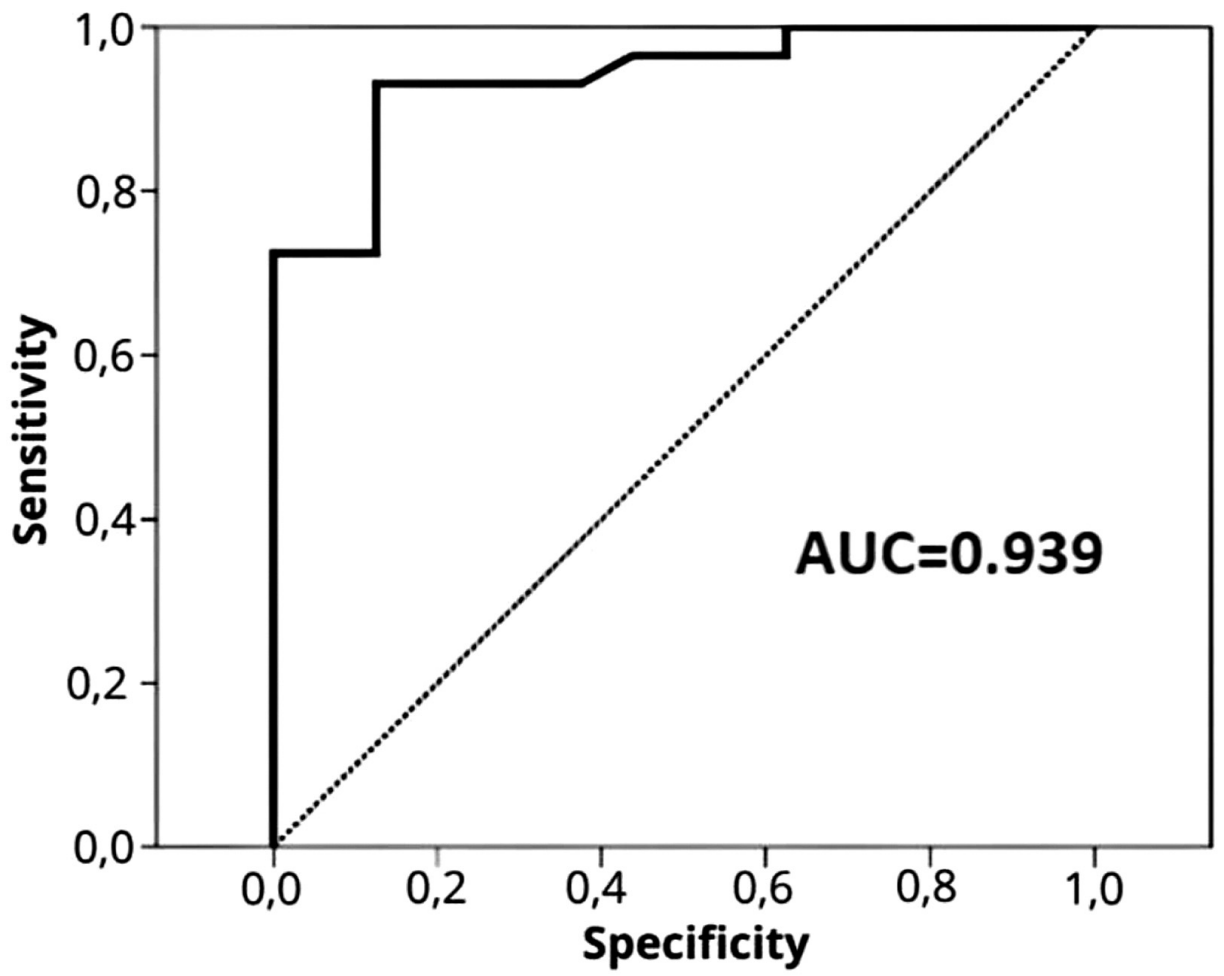
Receiver operating characteristic (ROC) curve of the adenoma-to-cerebellar peduncle (ACP) ratio for predicting PMA consistency. The area under the curve (AUC) is 0.939, indicating high accuracy.

Figure 3 illustrates the receiver operating characteristic (ROC) curve assessing the discriminatory performance of the ACP ratio derived in the present study. The ACP ratio on T2WI yielded an area under the curve (AUC) of 0.939 for the prediction of PMA consistency. Thresholds > 1.59 were strongly associated with soft PMAs, providing a sensitivity of 72.4% and a specificity of 100.0%, whereas thresholds < 1.27 were indicative of fibrous PMAs, with a sensitivity of 100.0% and a specificity of 37.5%, as summarized in Table 3 .

**Table 3:** Sensitivity and specificity of the adenoma-to-cerebellar peduncle ratio on T2-weighted imaging for predicting the consistency of pituitary macroadenomas

Adenoma-to-cerebellar peduncle ratio	Sensitivity (%)	Specificity (%)
0.900000	100.0	12.5
1.065000	100.0	25.0
1.190000	100.0	31.2
1.275000	100.0	37.5
1.310000	96.6	34.1
1.575000	72.4	6.6
1.590000	72.4	100.0
1.605000	65.5	100.0

**Figure 4:**
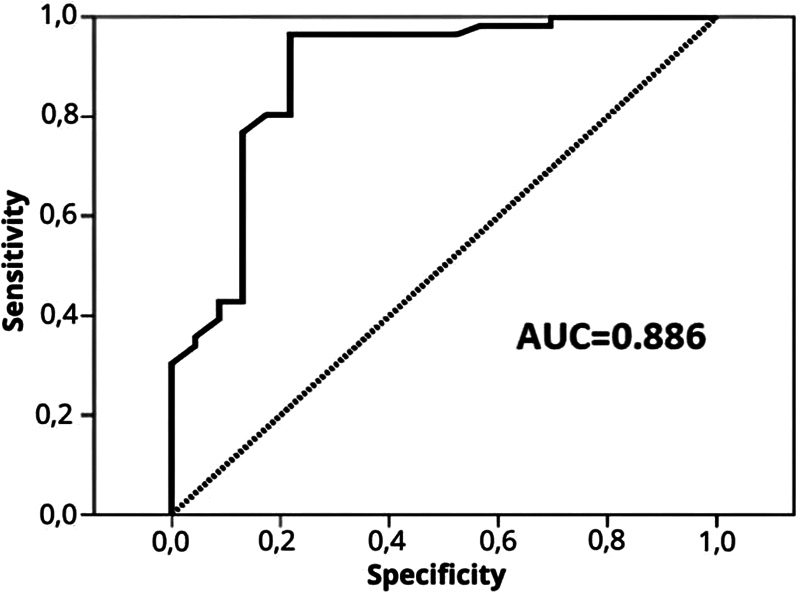
Combined receiver operating characteristic (ROC) curve analysis of the present study and Smith et al. 1 The combined area under the curve (AUC) is 0.886, highlighting the reliability of the ACP ratio across both datasets in predicting PMA consistency.

To compare our findings with existing literature, we utilized data from Smith et al. 1 to create a ROC curve. The AUC in our series was 0.939 (standard error [SE] = 0.034), while the AUC from Smith et al. 1 was 0.804 (SE = 0.080). The difference in AUC values between our series and the comparison study was 0.135 (SE = 0.087), which was not statistically significant ( *p* = 0.122). Consequently, a new ROC curve was prepared by combining data from both studies, yielding an AUC of 0.886 (SD = 0.46; 95% CI = 0.795–0.977) ( Figure 4 ). In the combined series, SI indices on T2WI > 1.79 were linked to soft PMAs, achieving a sensitivity of 30.4% and specificity of 100%. Meanwhile, SI indices on T2WI < 1.27 were associated with fibrous PMAs, with a sensitivity of 100% and specificity of 30.4%, as shown in Table 4 .

**Table 4:** Comparison of sensitivity and specificity for adenoma-to-cerebellar peduncle ratio on T2-weighted imaging in soft and fibrous pituitary macroadenomas between our series and that of Smith et al. ^1^

Adenoma-to-cerebellar peduncle ratio	Sensitivity (%)	Specificity (%)
0.930000	100	4.3
1.065000	100	21.7
1.190000	100	26.1
1.275000	100	30.4
1.310000	98.2	34.1
1.575000	80.4	86.2
1.590000	76.8	87
1.605000	73.2	87
1.790000	30.4	100
1.825000	26.8	100

## DISCUSSION

The present study corroborates the findings of Smith et al., demonstrating that the MRI ACP ratio is a reliable parameter for the preoperative prediction of PMA consistency. Our cutoff values ranged from 1.27 to 1.59. In our analysis, lower SI on MRI T2WI corresponded with fibrous tumors and aligned with classifications made by a neurosurgeon. Transsphenoidal surgery is recognized as a minimally invasive and safer method for the resection of pituitary adenomas. 1 3 4 5 6 7 8 9 However, it is primarily suitable for patients with soft PMAs, which facilitates complete tumor removal. Thus, preoperative assessment of pituitary adenoma consistency through neuroimaging is a critical consideration when planning surgical interventions.

The most significant studies aimed at predicting PMA consistency preoperatively utilized MRI T2WI or diffusion-weighted imaging (DWI) techniques. Pierallini et al. 16 demonstrated a notable correlation between DWI and MRI with apparent diffusion coefficient (ADC) values and tumor consistency in 22 PMA patients. A substantial correlation was found between tumor consistency and ADC values, SI ratio in DWI, SI ratio in T2WI, and collagen content percentage. However, other studies did not establish a significant correlation between DWI/ADC values and PMA consistency. 15 17 For instance, Suzuki et al. 15 were unable to identify any relationship between tumor consistency and ADC values in a prospective study involving 19 patients. They compared ADC values of soft consistency tumors with those of intermediate consistency tumors without evaluating fibrous tumors, which, coupled with their limited patient sample, may have hindered strong conclusions regarding the role of neuroimaging in predicting PMA consistency in their research.

A quantitative analysis correlating T2WI values with tumor consistency would yield more valuable insights than qualitative assessments. Previous studies addressing MRI features of pituitary adenomas have produced conflicting results concerning the prediction of tumor consistency. Iuchi et al. 11 noted that uniform low SI on T2WI was associated with firm pituitary adenomas. Conversely, other studies have indicated that an isointense appearance on T2WI was linked to firm tumors during surgical procedures 3 12 . Some authors 3 have proposed that PMA consistency can be anticipated based on T2WI, concluding that fibrous PMAs exhibited an isointense SI on T2WI compared with white matter. In contrast, Iuchi et al. 11 observed that fibrous PMAs had a hypointense SI on T2WI compared with white matter. Naganuma et al. 12 asserted that fibrous PMAs displayed isointense SI on T2WI when compared with surrounding brain tissue. Yang et al. 10 concluded that fibrous tumors exhibited a hypointense SI on T2WI, utilizing a ratio of tumor/white matter SI on T2WI. In contrast, other researchers 18 concluded that PMA consistency could not be reliably predicted using T2WI.

In our examination, 16 fibrous tumors were identified, 8 of which were homogeneous and 8 heterogeneous on T2WI. Additionally, 29 tumors were classified as soft, with 15 homogeneous and 14 heterogeneous. Based on this, we determined that tumor consistency could not be reliably predicted solely by MRI signal intensities, consistent with findings by Bahuleyan et al. 14

Given its adaptability, we opted to reanalyze T2WI using cutoff points. Among all tumors in the study, 6 fibrous tumors exhibited ratios < 1.3, while 21 soft tumors had ratios > 1.6. Tumors with ratios between 1.3 and 1.6 were variably classified as soft or fibrous. Nonetheless, none of the fibrous tumors had ratios > 1.6, and none of the soft tumors had ratios < 1.3. This indicates that these cutoff ratio values are effective, demonstrating high sensitivity and good specificity for determining tumor consistency, aligning with findings from Smith et al. 1

In the present investigation, tumors were categorized into two main groups: soft and fibrous PMAs. However, clinical practice acknowledges that tumor consistency can vary within these categories. Tumors with ACP ratios between 1.27 and 1.59 likely represent an intermediate group, exhibiting mixed characteristics that do not clearly align with either soft or fibrous classifications. This intermediate range may indicate variability in tumor composition or response to surgical techniques, thus requiring careful interpretation in clinical practice. Future studies should focus on further delineating the clinical relevance of this intermediate zone, which could refine preoperative planning and improve surgical outcomes.

A limitation of the present study is the lack of assessment of diffusion techniques for the evaluation of PMAs. Additionally, the study primarily focused on sellar imaging, without addressing the importance of complementing this with full brain imaging for a more comprehensive analysis of the ACP ratio. Future studies should consider integrating both T2-weighted imaging and diffusion-weighted imaging, as well as incorporating full brain imaging, to assess whether these combined approaches can further enhance preoperative tumor consistency prediction.

In conclusion, our study established a strong correlation between adenoma-to-cerebellar peduncle ratios and adenoma consistency. Additionally, it was demonstrated that utilizing the adenoma-to-cerebellar peduncle ratio is superior to relying solely on isolated SI on T2WI for determining PMA consistency. Furthermore, it was observed that ACP cutoff points < 1.3 exhibited high accuracy in predicting fibrous adenomas, while ratios > 1.6 demonstrated high accuracy for predicting soft adenomas.
